# Human Chemical Exposure from Background Emissions in the United States and the Implication for Quantifying Risks from Marginal Emission Increase

**DOI:** 10.3390/toxics9110308

**Published:** 2021-11-15

**Authors:** Dingsheng Li, Li Li

**Affiliations:** School of Public Health, University of Nevada, Reno, NV 89557, USA

**Keywords:** background emission, exposure modeling, dose–response relationship, human health risk assessment

## Abstract

The linear dose–response relationship has long been assumed in assessments of health risk from an incremental chemical emission relative to background emissions. In this study, we systematically examine the relevancy of such an assumption with real-world data. We used the reported emission data, as background emissions, from the 2017 U.S. National Emission Inventory for 95 organic chemicals to estimate the central tendencies of exposures of the general U.S. population. Previously published nonlinear dose–response relationships for chemicals were used to estimate health risk from exposure. We also explored and identified four intervals of exposure in which the nonlinear dose–response relationship may be linearly approximated with fixed slopes. Predicted rates of exposure to these 95 chemicals are all within the lowest of the four intervals and associated with low health risk. The health risk may be overestimated if a slope on the dose–response relationship extrapolated from toxicological assays based on high response rates is used for a marginal increase in emission not substantially higher than background emissions. To improve the confidence of human health risk estimates for chemicals, future efforts should focus on deriving a more accurate dose–response relationship at lower response rates and interface it with exposure assessments.

## 1. Introduction

Assessment of human health risks associated with exposure to chemicals released from activities of interest finds many applications in safeguarding the public interest. Prominent recent examples include the assessments of consumer exposure to 20+ disinfectants during the COVID-19 pandemic due to an elevated use of cleaning products [1], children’s exposure to 400+ chemicals of concern due to frequent contact with plastic toys [2], and consumer co-exposure to 700+ chemicals due to the daily use of consumer products [3]. Some of these assessments focus on the determination of whether harm is likely to happen or not. Examples include the comparison between external exposure with the reference dose in traditional noncancer risk assessments [4] or, more recently, the use of New Approach Methodologies comparing internal exposure with in vitro bioactivity data [1,5,6]. In traditional cancer risk assessments, exposure rates can also be converted to risks, with a predetermined toxicological factor, e.g., the cancer slope factor or unit risk [7]. When the exposure rate is coupled with a probability of risk to the population, assessment results can also be interpreted as health impact to the population in life cycle impact assessment [8].

In general, these assessments quantify health risk or impact by multiplying an environmentally relevant exposure with a fixed dose–response “slope” (i.e., the health impact resulting from a unit exposure dose). Such a slope is often derived from toxicological endpoints measured at high doses with high responses. Typical endpoints include the median effective dose (*ED_50_*) that corresponds to the population incidence response level of 50%, and, more recently, the 10% effective dose (*ED_10_*) recommended by the latest version of the life cycle impact assessment scientific consensus model USEtox [9]. Underlying this practice is an assumption of the linear dose–response relationship, in which the health effect (response) is independent of the exposure rate (dose) without a safe dosage (threshold). This linear assumption means that a marginal increase in the exposure rate always results in the same marginal increase in health effects. In other words, the resulting increment in health risk or impact is independent of the background exposure dose, i.e., the long-term baseline exposure level without impacts of the activity of interest.

While the linear dose–response relationship is intuitively simple in risk assessment, it may not necessarily represent the real mechanistic link between dose and response. In fact, the dose–response curve can be quite diverse: the literature is full of examples of the nonlinear, monotonic curves (i.e., the threshold model) and even nonmonotonic curves (i.e., the hermetic model) [10]. We can expect biases, i.e., the overestimation or underestimation of health risk and impact, when using a linear relationship to approximate the real nonlinear dose–response relationship. Notably, such biases can be remarkable if the environmentally relevant dose level diverges substantially from the toxicological dose from which the effect factor is derived (e.g., *ED_50_* or *ED_10_*). Indeed, a recent methodological paper for toxicity characterization of chemicals proposed using lower toxicological doses to derive such an effect factor to ensure its environmental relevance [9]. This bias issue can be even compounded when the background exposure dose, i.e., exposure resulting from existing sources of emission (background chemical emissions) other than the activity of interest, is taken into consideration, given that in a nonlinear dose–response curve, the increment in health risk or impact depends on not only the incremental exposure, but also the background exposure dose. In our earlier works, we showcased that the linear dose–response relationship may overestimate the risk when compared to the results from a nonlinear dose–response relationship hypothetically more representative of the real interplay between dose and response [11,12].

This work seeks to systematically address the extent to which a linear dose–response relationship is adequate for risk quantification when we consider a marginal increase in exposure dose over the background level. Specifically, we analyze the relationship between linear and nonlinear dose–response relationships to explore (i) whether the magnitude of exposure due to current background chemical emissions is so low that the linear extrapolation of effect factors derived from high-dose toxicological endpoints becomes less reasonable, (ii) whether, and in what situation, the use of a linear dose–response slope can reasonably approximate the nonlinear dose–response relationship, and (iii) how the increase in health impacts responds to incremental emissions over the background emissions in the nonlinear dose–response relationship. We also used reported background emission rates of 95 organic chemicals in the United States to construct realistic exposure levels and quantified the associated risks. This establishment of background exposure was then compared to the nonlinear dose–response relationship curve to determine at what magnitude marginal increase in emission could make certain linear dose–response relationship assumptions unreasonable.

## 2. Materials and Methods

### 2.1. General Approach

**Figure 1:**
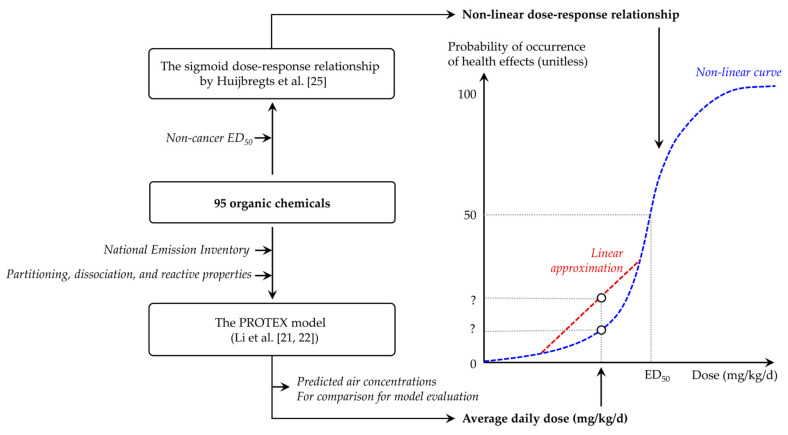
Schematic overview of the modeling approach in this study.

Figure 1 presents an overview of the approach used in this work. We used a comprehensive fate and exposure model named PROduction-To-EXposure (PROTEX) to predict the typical, environmentally relevant magnitude of human exposure due to current background chemical emissions in the United States (Section 2.3). To this end, we ran PROTEX with a steady-state assumption, given that the steady-state is recommended as a conservative representation of the maximal, reasonable level of long-term contamination and exposure in risk and impact assessments [13]. PROTEX requires partitioning, dissociation, and reactive properties as inputs (Section 2.2), and outputs an average daily dose (in mg_chemical_/kg_bodyweight_/*d*) as an estimate of human exposure to chemicals. On the other hand, we constructed a nonlinear dose–response relationship, which links a given dose (mg_chemical_/kg_bodyweight_/*d*) to the probability of the occurrence of health effects (unitless), based on an assumed sigmoid curve and noncancer *ED_50_*, which represents the effective dose causing a probability of noncancer health effects of 50% for lifetime exposure (Section 2.4). By placing the dot representing the modeled average daily dose on the constructed dose–response curve, we can estimate the probability of the occurrence of adverse health effects among the general American population caused by background emissions.

### 2.2. Chemical Data

We selected 95 structurally diverse organic chemicals for this case study, given that their nationally representative emission rates are available from the United States National Emission Inventory [14] and their toxicities are well documented in the USEtox toxicity database [8].

The most recent version of NEI (2017 NEI) included 291 pollutants from the on-road, nonroad, point, and nonpoint sources with detailed spatial information. We aggregated the reported emission rates (in kg/year) for the 95 organic pollutants across all sources and locations; these emission rates represent the status quo of pollutant emissions in the United States and are referred to as “background emissions” in this work. Since our work adopted the steady-state assumption, we assumed that the background emission rates do not undergo a remarkable change in time. Generally, this assumption is valid, as the reported emissions from NEI in 2011, 2014, and 2017 (Table S1) show that the median of the difference between the largest reported value is merely 23% of the average reported values in these three reporting years, albeit some larger variations do exist.

Meanwhile, we searched USEtox toxicity database (ver. 2.12) for noncancer toxicity effective doses (*ED_50_*) of the 95 organic chemicals. Originally, USEtox reports this value with a unit of kg_chemical_/lifetime. We converted this value to a unit of mg_chemical_/kg_bodyweight_/*d* to be consistent with our exposure prediction in this study, using a standard body weight of 70 kg and a lifetime of 70 years (25,550 days) from USEtox. Note that for respiratory inhalation and oral ingestion routes, *ED_50_* values may be different due to route-specific mechanisms. We therefore utilized route-specific *ED_50_*, whenever available, in subsequent assessments.

We gathered the partitioning and reaction properties of these 95 organic chemicals (Table 1) as inputs for environmental fate and exposure modeling. Overall, experimentally determined values collected from the literature were given the highest priority. When experimentally determined values were unavailable, we used quantitative structure–activity/property relationships (QSAR/QSPR) to make predictions from the molecular structure. Most chemicals were located well within the applicability domains of the QSAR/QSPR models used here.

The complete list of chemicals studied, along with their emission, toxicity, and physical–chemical properties can be found in Table S2 in the Supplementary Materials.

### 2.3. Exposure Prediction

Based on the NEI emission rates and gathered properties of the 95 organic chemicals, PROTEX calculated chemical concentrations in various compartments and organisms in an archetypal, generic subtropical North American environment, as well as the exposure of chemicals by a median American throughout the lifetime. The rationale, structure, and mathematical representation of PROTEX have been documented elsewhere [21,22]. The parameterization of the subtropical North American environment (including the geometry of each environmental compartment, intercompartmental mass exchange rates, meteorological and hydrological conditions, atmospheric hydroxyl concentration, etc.) and the median American (including anthropometrics, dietary patterns, drinking rate, etc.) is detailed in Li and Li [12]. PROTEX’s performance in predicting respiratory and oral exposure has previously been well-evaluated against monitoring and biomonitoring data of various chemicals [12,21,22,23,24].

PROTEX contains a regional environmental fate module describing the accumulation, transport, and transformation of chemicals between an urban, industrial area and a rural area where produces and livestock are grown. With this module, we calculated chemical concentrations in different environmental compartments (e.g., compartments of soil, freshwater, sediment, vegetation, impervious surfaces, etc., in the urban, industrial area; compartments of soil, freshwater, estuary water, sediment, vegetation, etc., in the rural area). For simplification, we assumed that the NEI atmospheric emissions went exclusively to the air compartment of the urban, industrial area before partitioning into other compartments and migration to the rural area along with atmospheric and riverine flows. While the modeled region accounted for <1% of the total area of the United States, we assumed that 1% of the national annual emissions occurred in this region, given that population and industries are disproportionately distributed in the subtropical part of the country. We acknowledge that this assumption may be associated with uncertainty; however, it led to a reasonable agreement between predicted and reported air concentrations from U.S. EPA’s National Air Toxics Assessment (NATA), and the linearity of PROTEX also allows scaling model outputs to any emission rate if a site-specific simulation is desired in future studies. PROTEX also quantified the uptake and accumulation of chemicals by aquatic (planktivorous and piscivorous fish) and terrestrial organisms (beef and dairy cattle, pigs, poultry, and vegetables) living in the modeled rural area. These organisms served as the foods (fish, beef, milk, pork, chicken, vegetables, etc.) for a modeled individual.

The modeled individual took in chemicals from the area without direct emissions through (i) oral ingestion, i.e., consuming contaminated food originating from the aquatic and terrestrial organisms, and (ii) respiratory inhalation, i.e., inhaling contaminated air and airborne particles. We simulated the average daily dose (mg_chemical_/kg_bodyweight_/*d*) of chemical uptake by the modeled individual at ages 3, 14, and 25.

### 2.4. Dose–response Relationships

We used a sigmoid dose–response relationship Equation (1) to convert a given average daily dose (*X_i_*, mg_chemical_/kg_bodyweight_/*d*) to the probability of the occurrence of adverse health effects (*PrHE*, unitless) [25]:

(1) $$PrHE_{non-linear}\left(X_{i}\right)=\int_{0}^{X_{i}}\frac{e^{-\frac{1}{2}{\left(\frac{\log\frac{X_{i}}{ED_{50,i}}}{\sigma_{log}}\right)}^{2}}}{\sigma_{log}\times \sqrt{2\pi }\times X_{i}\times \ln10}dX_{i}$$

The overall shape of this curve is determined by the chemical toxicity (*ED_50_*_,i_, mg_chemical_/kg_bodyweight_/*d*) and the spread in human toxicological susceptibility (*σ_log_*, unitless) with a central tendency of 0.26 [25]. Equation (1) indicates that, for any chemical, *PrHE* depends on the relativity of the average daily dose to *ED_50_* (i.e., the ratio of *X_i_* to *ED_50_*_,*i*_ in Equation (1)) instead of the absolute value of *ED_50_*. Therefore, we constructed a curve based on Equation (1) with the daily dose expressed as a fraction of *ED_50_*.

Here, we did not take the assumption that no risks will happen when exposure is below a certain threshold for noncancer effects, for reasons detailed in our earlier study [12]. In addition, effectively, exposure at low enough doses will generate negligible risks that are statistically equivalent to no risk, as shown in the results of this study.

To address the question of whether, and in what situation, the use of a linear dose–response slope can reasonably approximate the nonlinear dose–response relationship, we focused on a range of *PrHE* between 1/1,000,000 (i.e., smaller than which a health effect is considered to be negligible) and 10% (i.e., *ED_10_*; the upper limit of possible health risks associated with environmentally relevant exposure dose). This corresponds to the range of *X_i_* between ~0% and 46.4% of *ED_50_*. We segmented this range into several intervals with the purpose of constructing linear approximations of the nonlinear dose–response curve using the following steps. First, we took the marginal slope of the nonlinear dose–response curve, *S_1_*, at the starting point of the first interval, at which a dose of *X_1_* results in a probability of the occurrence of adverse health effects *PrHE*(*X_1_*), i.e.,

(2) $$S_{1}=\underset{\Delta X\to 0}{\lim}\frac{PrHE_{non-linear}\left(X_{1}+\Delta X\right)}{X_{1}+\Delta X}$$

Then, we can construct a linear dose–response curve for any dose *X* in the first interval Equation (3):

(3) $$S_{1}=\frac{PrHE_{linear,1}\left(X\right)-PrHE\left(X_{1}\right)}{X-X_{1}}$$

By rearranging Equation (3), the constructed linear dose–response curve gives *PrHE* for any dose *X* within the interval Equation (4):

(4) $$PrHE_{linear,1}\left(X\right)=S_{1}\times \left(X-X_{1}\right)+PrHE\left(X_{1}\right)$$

A reasonable linear approximation requires *PrHE*_nonlinear_ Equation (1) and *PrHE*_linear_ Equation (3) to be as close to each other as possible at any dose *X* within the interval. Obviously, as the dose increases further away from *X*_1_, the discrepancy between *PrHE*_nonlinear_ and *PrHE*_linear_ also increases. When the discrepancy increases to a factor of five, we considered the marginal slope *S*_1_ no longer to be appropriate for risk estimate and started the second interval. Likewise, we calculated a new marginal slope (*S_2_*) at this exposure (*X*_2_) as the starting point of the second interval. *PrHE_linear,_*_2_*(X)* was calculated similarly using Equation (3) based on *S_2_* and *X_2_* and, again, was compared with *PrHE*_nonlinear_*(X)* until a discrepancy of a factor of five was reached. This iterative process was then repeated until the entire range of *PrHE* between 1/1,000,000 and 10% was covered. The factor of five is based on a general understanding of the magnitude of uncertainties in toxicity and risk assessments. For instance, uncertainty factors for different aspects in deriving reference doses have a range of 1 to 10 [26]; comparisons between multiple toxicity endpoints also found the differences range from one to two orders of magnitude [8]. Thus, our choice of a factor of five represents a rather rigorous assumption.

In so doing, we segmented this range into several intervals. Next, we took the starting and ending points of each interval and extrapolate a fixed slope between these two points to derive a linear approximation of each interval. This was performed so that, when compared to the *PrHE* from the nonlinear dose–response curve, *PrHE* from these linear approximations would deviate even less than *PrHE* from the marginal slopes at starting points of intervals (less than a factor of two, see details in the Results section). Moreover, these extrapolated slopes between the starting and ending points of intervals will produce a higher, instead of lower, *PrHE*, which is often preferred in risk assessment, as such a more conservative estimate is more protective of human health. In addition, the starting point of the first interval was set to 0 to capture the whole range of exposure. This set of linear dose–response curves provides an intuitive approach to estimate *PrHE*.

### 2.5. Risk Quantification

Based on Equation (1), analytically, the risk is determined by the relative magnitude between daily dose and *ED*_50_, independent of their respective absolute values. When considering the combined risks from respiratory inhalation and oral ingestion exposure, we first determined this relativeness by expressing respiratory inhalation and oral ingestion exposures as fractions of their respective *ED*_50_. These fractions were then added together as the combined relative exposure to *ED*_50_:

(5) $$X_{\%ED50,i}=\frac{X_{inh,i}}{ED_{50,inh,i}}+\frac{X_{ing,i}}{ED_{50,ing,i}}$$

Since the estimated risk is only determined by the relative magnitude between exposure and *ED*_50_, we can set *ED*_50_ to 1 and calculate the total risk with the following equation modified from Equation (1):

(6) $$PrHE\left(X_{inh,i},X_{ing,i}\right)=\int_{0}^{X_{\%ED50,i}}\frac{e^{-\frac{1}{2}{\left(\frac{\log X_{\%ED50,i}}{\sigma_{log}}\right)}^{2}}}{\sigma_{log}\times \sqrt{2\pi }\times X_{\%ED50,i}\times \ln10}dX_{\%ED50,i}$$

Furthermore, we can apply the same principle to calculate the total relative exposure to *ED*_50_ for all 95 chemicals and the associated risk (*PrHE_total_*) with the following two equations:

(7) $$X_{\%ED50,total}=\sum X_{\%ED50,i}$$

(8) $$PrHE_{total}=\int_{0}^{X_{\%ED50,total}}\frac{e^{-\frac{1}{2}{\left(\frac{\log X_{\%ED50,total}}{\sigma_{log}}\right)}^{2}}}{\sigma_{log}\times \sqrt{2\pi }\times X_{\%ED50,total}\times \ln10}dX_{\%ED50,total}$$

## 3. Results

### 3.1. Predicted Human Exposures and Environmental Concentrations

**Figure 2:**
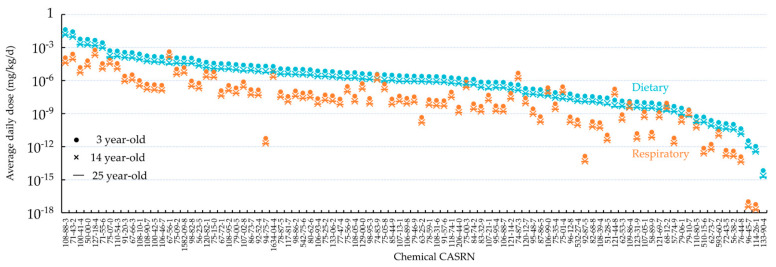
Average daily doses (ingestion in blue and inhalation in orange) of 95 organic chemicals for 3-, 14-, and 25-year-old individuals predicted in this study based on background emission rates from NEI, ranked by the average daily oral dose for age 3.

Figure 2 shows the predicted average daily doses for respiratory and oral exposure of the general American population to 95 organic chemicals. For both respiratory and oral exposures, the average daily dose varied over 13 orders of magnitude between chemicals. Specifically, the average daily oral dose was highest for toluene (CASRN 108-88-3), whereas the average daily respiratory dose was highest for methanol (CASRN 67-56-1). Oral ingestion is more, up to a factor of 2 × 10^7^ times, important than respiratory inhalation for most chemicals, with exceptions of methanol (CASRN 67-56-1), methyl chloride (CASRN 74-87-3), 1,3-butadiene (CASRN 106-99-0), vinyl chloride (CASRN 75-01-4), triethylamine (CASRN 121-44-8), and *N*,*N*-dimethylformamide (CASRN 68-12-2). For all the 95 organic chemicals, 3-year-old children possess ~3 times higher average daily doses, relative to 14-year-old teenagers and 25-year-old adults.

To evaluate the fidelity of the PROTEX predictions, we selected chemicals that demonstrated the top 10 highest estimated risks and compared their air chemical concentrations predicted by PROTEX with predictions from the latest NATA [27]. Here, NATA predicted air concentrations at the census tract level across the country, using air quality models such as CMAQ and AERMOD supplied with modified NEI data. We aggregated the census track-specific estimates and calculated the 95% confidence intervals (defined by 2.5th and 97.5th percentiles) as reasonable estimates of the typical level of contamination in the United States (Table S3). Overall, for eight of the 10 chemicals, the PROTEX predictions fell well within the 95% confidence intervals of NATA predictions, with a discrepancy between the PROTEX predictions and NATA’s means generally smaller than a factor of 10. Of particular attention is that PROTEX underpredicted the concentration of carbon tetrachloride by a factor of 180. Such underprediction is because PROTEX assumes that emissions within the modeled region are the predominant source of contamination therein, which may not be the case for carbon tetrachloride, because it is a global persistent pollutant capable of long-range transboundary atmospheric transport, and the emissions within the U.S. accounted for merely 8% of the worldwide total [28].

**Figure 3:**
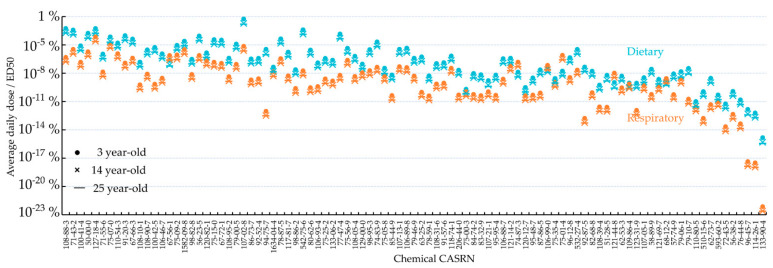
The ratio of the average daily dose to *ED*_50_ for 95 organic chemicals (ingestion in blue and inhalation in orange) for 3-, 14-, and 25-year-olds predicted in this study based on background emission rates from NEI; the chemicals are ranked in the same order as in Figure 2.

Figure 3 shows that the ratio of the predicted average daily dose to *ED*_50_ varies by 12 orders of magnitude for oral exposure and 17 orders of magnitude for respiratory exposure between chemicals. This ratio is the highest for acrolein (CASRN 107-02-8; ratio = 0.55%) regarding oral exposure and for tetrachloroethylene (CASRN 127-18-4; ratio = 0.053%) regarding respiratory exposure. For all the 95 chemicals investigated here, the predicted average daily dose accounts for less than 1% of the corresponding *ED_50_*. For most chemicals, the ratio is higher for oral ingestion than respiratory inhalation. Mirroring the age-dependence of average daily dose (Figure 2), 3-year-old children possess higher ratios of average daily dose to *ED*_50_, compared to 14-year-old teenagers and 25-year-old adults.

### 3.2. Linear Approximations along the Nonlinear Dose–response Curve

**Figure 4:**
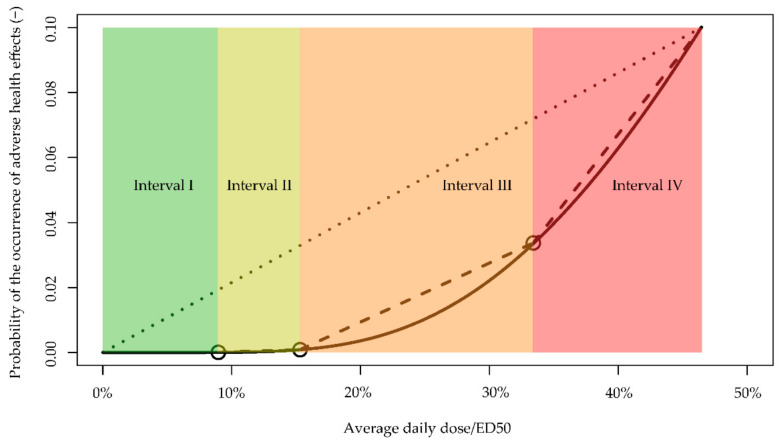
Nonlinear dose–response curve (solid line), approximated linear slopes for four intervals (dashed lines), and extrapolated slope based on *ED*_10_ (dotted line). Circles are starting/end points for the intervals.

Within the range of *PrHE* between 1/1,000,000 and 10%, we identified four intervals (I–IV) (Figure 4): The first covers exposure from 0 to 9.0% of *ED_50_*, the second from 9.0% to 15.3% of *ED*_50_, the third from 15.3% to 33.4% of *ED*_50_, and the fourth from 33.4% to 46.4% of *ED*_50_. Within each interval, we consider that a linear dose–response relationship can be a simpler implementation for characterizing health risks, compared to the nonlinear dose–response relationship (dash lines in Figure 4).

Using the linear approximation, the estimated *PrHE* will be higher compared with results from the nonlinear dose–response relationship, with the largest difference near the beginning of each internal. These differences are the largest for interval I, as with low exposures, the nonlinear dose–response relationship will produce exceedingly small values of risks. The difference becomes smaller than an order of magnitude when exposure is higher than 6.3% of *ED*_50_ (at which point the nonlinear dose–response relationship suggests a risk of 1.94 × 10^−6^). For intervals II, III, and VI, the differences in estimated *PrHE*s between the two relationships are no more than 175%, 176%, and 10%, showing relatively good agreement. In contrast, a fixed linear slope based on *ED*_10_ (dotted line in Figure 4) will produce much higher risk estimates until exposure reaches interval IV.

### 3.3. Exposures and Risks from Background Emissions

Table 2 shows the combined ingestion and inhalation exposures as fractions of *ED*_50_ and associated probability of occurrence of health effects (*PrHE*) of the top 10 chemicals. The full result for 95 organic chemicals can be found in Table S4 in the Supplementary Materials.

Despite being orders of magnitude higher than others, the highest *PrHE* estimated in this study, which, for a 3-year-old exposed to acrolein, was still negligible (<1 in a million). The total relative exposures to *ED*_50_ from all 95 organic chemicals analyzed were 0.00823, 0.00282, and 0.00242 for a 3-year-old, a 14-year-old, and a 25-year-old, respectively. The risks estimated from these exposure levels were 5.43 × 10^−16^, 5.23 × 10^−23^, and 4.16 × 10^−24^ (Table S4). These exposure levels are still within the first interval on the nonlinear dose–response relationship curve.

### 3.4. Application of Linear Approximations for Estimating Risks from Incremental Emissions

Based on our analysis of the dose–response relationship and the background risks from NEI emissions, we propose an intuitive way to calculate *PrHE* associated with an incremental exposure, which results from an incremental emission rate due to the activity of interest:

Step 1: For any of the 95 chemicals included in our analysis, search Table S4 for the baseline average daily dose (expressed as a fraction of *ED*_50_) resulting from the background emission rate.

Step 2: Calculate the average daily dose due to an incremental emission rate (expressed as a fraction of *ED_50_*) using exposure modeling, e.g., PROTEX.

Step 3: Add these two daily doses to give a total average daily rate (*X*), assuming the average daily dose scales linearly to the emission rate.

Step 4: Search Table 3 to locate the interval (bracket) that *X* falls in, and calculate *PrHE* using the following equation:

*PrHE* (*X*) = *S* × (*X* − *X*_0_) + *R*_0_ (9)

For example, acrolein has a background exposure for a 3-year-old of 0.00547 of its *ED*_50_ (Table 2) with a national background emission of 10.1 million kg (Table S1). If we assume that an incremental emission of acrolein occurs for 101 million kg and exposure scales linearly to emission, the total exposure as a fraction of *ED*_50_ (*X*) would be 0.00547 (background) + 0.0547 (incremental) = 0.0602. This is still within Interval I. Thus, the total risk would be 1.87 × 10^−7^. Suppose a higher incremental emission occurs for 505 million kg of acrolein: assuming exposure scales linearly with emission again, the total exposure as a fraction of *ED*_50_ due to background and incremental would then be 0.279. This is within Interval II. Thus, the total risk would be 2.35 × 10^−2^.

Theoretically, the assessment of health risks and impacts should be based on the total exposure, which includes not only the incremental exposure due to the activity of interest but also the exposure caused by background emissions. However, the above calculations (Table 2 and Table S4) show that the background emissions likely pose negligible risks to the general population, and that the exclusion of background emissions is unlikely to have an influential effect on the results. Therefore, for chemicals without information on background emissions and exposures, the risks associated with known incremental emissions alone should not differ substantially from risks calculated with background emissions.

## 4. Discussion

This study provided four linear approximations of the nonlinear dose–response relationship, which offer a potentially more accurate, yet still intuitive, approach to quantify risks from exposures to chemicals. Our approach will not produce results more than an order of magnitude higher than that from the nonlinear dose–response relationship for exposure higher than 6.3% of *ED*_50_. By contrast, using an extrapolated linear slope from no exposure/risk to an exposure of *ED*_10_ [9] (dotted line in Figure 4) could produce results at 7000 times higher for the same range of exposure. Notably, we provided a predetermined estimate of the linear dose–response slope for the interval where exposure resulting from background emission is relevant (Interval I in Table 3). Such linearity eases the assessment of the health risks and impacts of the environmentally relevant dose of exposure. Given that exposure levels from background emissions are far below the starting point of Interval II, we expect that the risks associated with a marginal increase in emission for any of the 95 chemicals can still be reasonably quantified by a linear dose–response relationship with the predetermined linear slope, as long as the increment is less than orders of magnitude of corresponding background emission. The only exception is for exposure of 3-year-old children to acrolein, in which the slope of Interval I would be no longer valid, and that of Interval II would be needed if the marginal increase in emission exceeds about 15 times the background emission.

In this study, we predicted the average daily doses of 95 organic chemicals by Americans aged 3, 14, and 25 through oral ingestion and respiratory inhalation. The predictions are believed to be representative of the central tendencies of Americans’ chemical exposures, given that the PROTEX-predicted air chemical concentrations agree well with the national means estimated by NATA. Note that NATA predicts only human respiratory inhalation of chemical contamination in the air, while PROTEX additionally considers contamination in the multimedia environment and the aggregate human exposure through multiple routes. Notably, Figure 2 shows that, compared to respiratory inhalation, oral ingestion made an over 300 times higher contribution to the total average daily dose. The ratio of average daily dose to *ED*_50_ was up to 2 × 10^7^ times higher for oral ingestion than respiratory inhalation (Figure 3). These comparisons indicate that diet is also a relevant route responsible for human exposure to chemicals released into air, in particular when chemicals are relatively bioaccumulative due to hydrophobicity and resistance to degradation [29]. Focusing on respiratory inhalation may substantially underestimate the overall human exposure to certain chemicals.

The work shows that children typically had higher daily doses of chemicals compared to teenagers and adults. This is mostly because children have high rates of food ingestion, relative to their small body weights, to support fast growth and development. This finding implies disproportionately higher health impacts on children even if they live in a similarly contaminated environment with parents.

A common feature found for the 10 chemicals with the highest estimated risks is that they (i) share high emission rates, given that seven of them also were on the list for top 20 in emission rates, and (ii) possess high toxicity, manifested by relatively low *ED*_50_ values for respiratory/oral exposure, or both. A typical case is for hexachlorocyclopentadiene, which was ranked 76th in emission rates but 7th in the estimated risk due to its high toxicity for inhalation (ranked 4th). Despite the fact that the notion that higher emission will result in higher risk seems intuitive, the Pearson correlation coefficient between the rankings of emission amounts and estimated risks was a moderate 0.59. This echoes our earlier finding that chemical tonnage plays a limited role in determining health risks [30]. Highlighting this is the case of methanol, the top emitted chemical with over 2 billion kg reported emission—more than the other 94 chemicals’ emission amounts combined. It was only ranked 34th in estimated risk because of low toxicity (90th for both inhalation and ingestion). Chemical properties also impact the estimated risks: chemicals that are highly hydrophilic (i.e., associated with a low octanol–water partition coefficient *K*_OW_), highly volatile (i.e., associated with a high octanol–air partition coefficient *K*_OA_), and/or liable to reaction in the environment or organisms (i.e., associated with short biodegradation or biotransformation half-lives) tend to be minimally taken up by humans, especially through diet [24,29]. A complete ranking for emission rate, toxicity values, and estimated risks for the 95 organic chemicals can be found in Table S5 in the Supplementary Materials. The complex relationship between emission amount and risk could have implications when designing epidemiology studies and interpreting environmental monitoring data for risk if emission amount was the primary, if not only, factor considered [31,32,33,34,35]. This complex relationship also brings into question the suitability of using chemical tonnage (e.g., production, trade, or emission volume) alone in chemical regulation and management.

Based on methods proposed in this study, all background emissions only resulted in minimal risks, which is consistent with findings of the most recent NATA [36]. However, it should be noted that this conclusion depends on the assumption of homogenous distribution of the pollutant and exposure over a large area. In reality, the distribution of pollution can be highly concentrated in certain areas (e.g., occupational settings near polluting processes, the proximity of a factory emitting the pollutant, downwind of a wildfire, area surrounding roads with heavy traffic) and result in higher exposure and risk. Therefore, the numeric results for risk estimates in this study should not be interpreted, as environmental pollution is irrelevant to public health. Environmental and exposure models that can produce finer spatial resolution would provide more relevant data to estimate risks. Indeed, NATA also indicated that 100 census tracts (out of more than 70 thousand) could experience some noncancer respiratory risks from emissions reported by NEI [36].

It should be noted that the numeric values of health risks in this study were dependent on Equation (1) describing the nonlinear dose–response relationship, which was published more than 15 years ago. To our knowledge, no refined nonlinear equations that can similarly calculate the risk based on exposure have been proposed since. Recently, a sophisticated model, APROBA, which produces probabilistic reference doses for more than a thousand chemicals, has been developed [37,38]. However, the output from APROBA is more qualitative since the reference dose is an exposure threshold where, if not exceeded, no risk is expected. It is therefore challenging to deduce quantitative risk information from a given exposure with APROBA. To improve the confidence in risk quantification results, better fundamental knowledge and computational models are needed in this regard. This is especially true for estimating risks from low-dose exposure, which has been a challenge in risk assessment [39,40]. Based on this study, exposures resulting from background emissions are likely in the low-dose range and will stay in this range unless incremental emission causes an increase of orders of magnitude higher. Therefore, it is vital to better understand how to characterize human risks for low-dose exposure, which the development of New Approach Methodologies could offer assistance in [1,41,42,43,44].

In addition, in this study, we assumed homogenous exposure among individuals in the same age groups and toxicological susceptibility for all individuals across the population. In our previous study, we demonstrated that if interindividual variability was considered, the health impacts (the number of cases of diseases) for the population were dominated by the subpopulation with high exposure and toxicological susceptibility. The total fraction of the population having health effects would also increase substantially [12]. The largest uncertainty involved in quantifying interindividual variability risks is the lack of robust data and models for toxicological susceptibility for different chemicals. Certain New Approach Methodologies that could provide more insight on this issue have been developed [45,46,47,48]. It is our opinion that further research in this area is needed to enable more robust and population-relevant risk quantification.

## 5. Conclusions

This study presented an approach to linearly approximate the nonlinear dose–response relationship without creating substantial deviation in results when used for risk assessment. The dose-based linear approximations can potentially increase the accuracy of risk assessments compared to the conventional approach based on the linear dose–response relationship extrapolated from a fixed toxicity metric. The approach was applied to background pollutant emissions in the United States, yielding good agreement between our results and those from more sophisticated models. While the risks based on background emissions in the United States are minimal, it should be noted that certain microenvironments within the United States and other regions that have higher emissions could still pose risks at a concerning level. Future studies are needed to improve our understanding of the actual dose–response relationship for various chemicals, as well as interindividual variances in susceptibility, which will advance more precise human health risk assessments.

## Figures and Tables

**Table 1:** Properties of the 95 organic chemicals examined in this study.

Property	Experimentally Determined Value (EDV)	Model Predicted Value (MPV)
Equilibrium octanol–water partition coefficient (*K*_OW_)	91 chemicals with EDVs from the PHYSPROP database	4 chemicals with MPVs as consensus values (geometric means) of predictions made with OPERA (all within the applicability domain or AD) [15] and the KOWWIN module in EPI Suite [16] (all within the AD ^(a)^).
Equilibrium octanol–air partition coefficient (*K*_OA_)	N.A.	95 chemicals with MPVs as consensus values (geometric means) of predictions made with OPERA (89 chemicals within the AD) and the KOAWIN module in EPI Suite (92 chemicals within the AD).
Dissociation rate constant (p*K*_a_ and p*K*_b_)	46 chemicals do not dissociate in the environment (neither acids nor bases)	49 ionogenic organic chemicals with MPVs predicted by OPERA (all within the AD).
Atmospheric hydroxylation rate constant (k_(OH)_)	65 chemicals with EDVs from the PHYSPROP database	30 chemicals with MPVs as consensus values (geometric means) of predictions made with OPERA (22 chemicals within the applicability domain or AD) and the AOPWIN module in EPI Suite (25 chemicals within the AD).
Biodegradation rate constant ^(b)^	11 chemicals with EDVs collected by Arnot et al. [17]	84 chemicals with MPVs as consensus values (geometric means) of predictions made with OPERA (39 chemicals within the AD), the BioHCWIN module in EPI Suite (for the 15 hydrocarbons only), and estimates converted using the empirical relationships in Arnot et al. [17] based on the probabilities of primary degradation predicted by the BIOWIN module in EPI Suite.
Biotransformation rate constant in fish (normalized to 10 g) ^(c)^	N.A.	All chemicals with MPVs as consensus values (geometric means) of predictions made with OPERA (89 chemicals within the AD) and IFS-QSAR [18] (81 chemicals within the AD).
Biotransformation rate constant in mammals (including humans) (normalized to 70 kg) ^(d)^	11 chemicals with EDVs collected by Arnot et al. (2014)	84 chemicals with MPVs predicted by IFS-QSAR [19] (67 chemicals within the AD).

Note: ^(a)^ EPI Suite does not provide explicit information on the applicability domain of each module. Instead, it suggests that a prediction is “less reliable” if it (i) is outside the range of experimental values in the training set, (ii) is outside the range of molar mass of chemicals in the training set, (iii) has more instances of a given fragment than the maximum for all training set compounds of functional groups, and/or (iv) contains structural features not represented in the training set. We define that a prediction is outside the applicability domain if it meets at least one of these conditions. ^(b)^ Rate constants of degradation in water, soil, and sediment were assumed to be 1, 2, and 10 times slower than the biodegradation rate constant, based on a generic empirical relationship by Fenner et al. [20]. ^(c)^ Used with an allometric correction for body weight for all aquatic organisms modeled in PROTEX. ^(d)^ Used with an allometric correction for body weight for all mammals and avian species modeled in PROTEX.

**Table 2:** Combined ingestion and inhalation exposure as a fraction of *ED_50_* and estimated probability of occurrence of health effects (*PrHE*) for 10 chemicals with top *PrHE*, ranked in order.

CASRN	Chemical Name	Average Daily Dose to *ED*_50_ Ratio			PrHE		
		3-Year-Old	14-Year-Old	25-Year-Old	3-Year-Old	14-Year-Old	25-Year-Old
107-02-8	Acrolein	5.47 × 10^−3^	1.86 × 10^−3^	1.55 × 10^−3^	1.64 × 10^−18^	4.08 × 10^−26^	1.56 × 10^−27^
127-18-4	Tetrachloroethylene	5.96 × 10^−4^	2.07 × 10^−4^	1.90 × 10^−4^	1.25 × 10^−35^	7.08 × 10^−46^	9.20 × 10^−47^
108-88-3	Toluene	5.45 × 10^−4^	1.89 × 10^−4^	1.74 × 10^−4^	1.91 × 10^−36^	8.34 × 10^−47^	1.05 × 10^−47^
542-75-6	1,3-Dichloropropene	3.89 × 10^−4^	1.35 × 10^−4^	1.24 × 10^−4^	1.33 × 10^−39^	2.16 × 10^−50^	2.43 × 10^−51^
71-43-2	Benzene	3.67 × 10^−4^	1.27 × 10^−4^	1.17 × 10^−4^	3.61 × 10^−40^	4.93 × 10^−51^	5.52 × 10^−52^
50-00-0	Formaldehyde	1.72 × 10^−4^	5.89 × 10^−5^	5.11 × 10^−5^	7.75 × 10^−48^	8.03 × 10^−60^	1.66 × 10^−61^
77-47-4	Hexachlorocyclopentadien	1.38 × 10^−4^	4.79 × 10^−5^	4.39 × 10^−5^	3.47 × 10^−50^	2.69 × 10^−62^	2.47 × 10^−63^
91-20-3	Naphthalene	9.45 × 10^−5^	3.28 × 10^−5^	3.01 × 10^−5^	2.40 × 10^−54^	6.07 × 10^−67^	5.10 × 10^−68^
56-23-5	Carbon Tetrachloride	8.69 × 10^−5^	3.02 × 10^−5^	2.77 × 10^−5^	2.74 × 10^−55^	5.49 × 10^−68^	4.48 × 10^−69^
75-07-0	Acetaldehyde	8.40 × 10^−5^	2.84 × 10^−5^	2.33 × 10^−5^	1.12 × 10^−55^	8.97 × 10^−69^	2.65 ×10^−71^

**Table 3:** Risk quantification using approximated linear dose–response relationship.

Interval	Dose as a Fraction of *ED*_50_ (*X*/*ED*_50_)	Base Dose (*X_0_*)	Slope (*S*)	Base *PrHE* (*R_0_*)
I	(0, 0.090)	0	0.00031	0
II	(0.090, 0.153)	0.090	0.013	0.000028
III	(0.153, 0.334)	0.153	0.18	0.00085
IV	(0.334, 0.464)	0.334	0.51	0.034

## Data Availability

Publicly available datasets were analyzed in this study. The 2017 NEI data can be found at https://www.epa.gov/air-emissions-inventories/2017-national-emissions-inventory-nei-data (accessed on 23 July 2021); the toxicity data can be found in USEtox 2.12 package at https://usetox.org/model/download (accessed on 23 July 2021); the EPISuite 4.11 tool can be found at https://www.epa.gov/tsca-screening-tools/epi-suitetm-estimation-program-interface (accessed on 1 May 2021); the OPERA tool can be found at https://github.com/kmansouri/OPERA (accessed on 1 May 2021); the PROTEX model can be found at https://lilienv.weebly.com/protex.html (accessed on 1 May 2021).

## Supplementary Materials

The following are available online at https://www.mdpi.com/article/10.3390/toxics9110308/s1, Table S1: Emission amounts reported by NEI in 2011, 2014, and 2017 for the 95 organic chemicals analyzed in this study, Table S2: Chemical analyzed and relevant data used in this study, Table S3: Comparison between PROTEX predicted air concentration and predictions from U.S. EPA’s National Air Toxics Assessment, Table S4: Exposure as a fraction to ED_50_ and estimated risk for the 95 organic chemicals analyzed, Table S5: Rankings for emissions, *K*_OW_, toxicity values, and estimated risks for 95 organic chemicals analyzed in this study.
