# Supplementary material for: Human Chemical Exposure from Background Emissions in the United States and the Implication for Quantifying Risks from Marginal Emission Increase
Source: Toxics. 2021 Nov 15;9(11):308. doi: 10.3390/toxics9110308 (PMC8621763; doi:10.3390/toxics9110308)
Supplement: Supplementary file 1 [file toxics-09-00308-s001.zip › toxics-1401520-supplementary.pdf]

toxics

MDPI

Supplementary Materials: Human chemical exposure from background emissions in the United States and the implication for quantifying risks from marginal emission increase

Dingsheng and Li Li

Table S1. Emission amounts reported by NEI in 2011, 2014, and 2017 for the 95 organic chemicals analyzed in this study.

CASRN	Name	Total emission (kg)[a]		
		2011	2014	2017
100-41-4	Ethyl Benzene	7.44E + 07	7.00E + 07	5.36E + 07
100-42-5	Styrene	1.91E + 07	2.35E + 07	2.14E + 07
106-46-7	1,4-Dichlorobenzene	1.65E + 06	1.15E + 06	2.84E + 05
106-88-7	1,2-Epoxybutane	8.45E + 03	1.61E + 04	9.40E + 03
106-89-8	Epichlorohydrin	3.55E + 04	3.49E + 04	5.60E + 04
106-93-4	Ethylene Dibromide	2.05E + 04	1.72E + 04	1.99E + 04
106-99-0	1,3-Butadiene	2.12E + 07	1.94E + 07	1.51E + 07
107-02-8	Acrolein	1.02E + 07	1.17E + 07	1.01E + 07
107-05-1	Allyl Chloride	2.28E + 04	1.41E + 04	1.28E + 04
107-13-1	Acrylonitrile	3.18E + 05	3.32E + 05	2.65E + 05
107-21-1	Ethylene Glycol	6.22E + 07	6.63E + 07	4.11E + 07
108-05-4	Vinyl Acetate	7.77E + 05	7.32E + 05	5.95E + 05
108-10-1	Methyl Isobutyl Ketone	2.54E + 07	8.42E + 06	8.03E + 06
108-31-6	Maleic Anhydride	1.33E + 05	1.26E + 05	1.19E + 05
108-39-4	m-Cresol	6.34E + 03	8.52E + 03	9.87E + 03
108-88-3	Toluene	5.67E + 08	4.33E + 08	3.48E + 08
108-90-7	Chlorobenzene	8.16E + 05	7.37E + 05	4.31E + 05
108-95-2	Phenol	8.43E + 06	9.33E + 06	9.73E + 06
109-86-4	Ethylene Glycol Methyl Ether	3.03E + 04	1.91E + 05	1.79E + 05
110-54-3	Hexane	2.44E + 08	1.12E + 08	9.19E + 07
110-80-5	Cellosolve Solvent	2.86E + 04	1.34E + 04	2.99E + 03
114-26-1	Propoxur	7.35E + 00	1.51E + 00	1.60E-01
117-81-7	Bis(2-Ethylhexyl)Phthalate	1.58E + 05	1.95E + 05	1.95E + 05
118-74-1	Hexachlorobenzene	1.18E + 03	1.44E + 03	1.24E + 03
120-12-7	Anthracene	2.43E + 05	2.00E + 05	1.75E + 05
120-82-1	1,2,4-Trichlorobenzene	9.87E + 04	1.05E + 05	1.01E + 05
121-14-2	2,4-Dinitrotoluene	4.33E + 03	7.08E + 03	6.15E + 03
121-44-8	Triethylamine	4.81E + 05	1.82E + 05	1.70E + 05
121-69-7	N,N-Dimethylaniline	8.14E + 03	6.36E + 03	6.81E + 03
123-31-9	Hydroquinone	2.30E + 04	2.46E + 04	3.89E + 04
127-18-4	Tetrachloroethylene	1.11E + 07	3.60E + 06	7.53E + 06
129-00-0	Pyrene	4.47E + 05	3.64E + 05	4.72E + 05
133-06-2	Captan	1.01E + 02	2.89E + 05	2.27E + 05
133-90-4	Chloramben	1.02E + 00	3.00E − 02	2.00E − 02
1582-09-8	Trifluralin	2.20E + 06	2.29E + 06	2.81E + 06
1634-04-4	Methyl Tert-Butyl Ether	6.29E + 05	3.14E + 05	2.10E + 05
206-44-0	Fluoranthene	4.23E + 05	3.29E + 05	2.84E + 05
50-00-0	Formaldehyde	1.02E + 09	9.98E + 08	6.56E + 08
510-15-6	Chlorobenzilate	1.45E + 00	1.94E + 01	2.18E + 01
51-28-5	2,4-Dinitrophenol	6.45E + 03	1.26E + 03	6.04E + 03
532-27-4	2-Chloroacetophenone	3.08E + 03	2.69E + 03	6.65E + 02
542-75-6	1,3-Dichloropropene	2.75E + 06	1.74E + 06	1.91E + 05
56-23-5	Carbon Tetrachloride	9.73E + 04	9.25E + 04	1.70E + 05

56-38-2	Parathion	1.36E + 00	1.37E + 01	1.34E + 01
57-74-9	Chlordane	1.84E + 01	1.41E + 01	5.56E + 00
58-89-9	1,2,3,4,5,6-Hexachlorocy	3.04E + 02	1.99E + 00	2.83E + 01
593-60-2	Vinyl Bromide	7.74E + 01	7.09E + 01	4.64E + 01
62-53-3	Aniline	7.64E + 04	8.37E + 04	6.45E + 04
62-73-7	Dichlorvos	7.33E + 02	4.35E + 02	2.51E + 01
63-25-2	Carbaryl	1.77E + 04	3.17E + 05	3.53E + 05
67-56-1	Methanol	4.09E + 09	3.52E + 09	2.03E + 09
67-66-3	Chloroform	7.10E + 05	7.29E + 05	1.01E + 06
67-72-1	Hexachloroethane	7.12E + 04	4.92E + 04	3.36E + 04
68-12-2	N,N-Dimethylformamide	1.27E + 05	1.16E + 05	1.02E + 05
71-43-2	Benzene	1.64E + 08	1.49E + 08	1.21E + 08
71-55-6	Methyl Chloroform	2.06E + 07	1.65E + 07	9.80E + 06
72-43-5	Methoxychlor	2.18E + 01	1.43E + 01	6.56E + 00
74-83-9	Methyl Bromide	5.59E + 06	4.15E + 06	9.42E + 05
74-87-3	Methyl Chloride	7.75E + 05	1.99E + 06	1.26E + 06
75-00-3	Ethyl Chloride	1.71E + 05	2.40E + 05	2.38E + 05
75-01-4	Vinyl Chloride	3.21E + 05	3.86E + 05	3.87E + 05
75-05-8	Acetonitrile	3.93E + 05	4.53E + 05	7.19E + 05
75-07-0	Acetaldehyde	7.18E + 08	6.95E + 08	4.47E + 08
75-09-2	Methylene Chloride	7.92E + 06	6.47E + 06	3.15E + 06
75-15-0	Carbon Disulfide	4.31E + 06	4.64E + 06	4.13E + 06
75-25-2	Bromoform	3.07E + 04	3.10E + 04	1.60E + 04
75-35-4	Vinylidene Chloride	3.36E + 04	2.76E + 04	1.70E + 04
75-56-9	Propylene Oxide	3.57E + 05	2.87E + 05	2.49E + 05
76-44-8	Heptachlor	5.34E + 00	4.32E + 00	2.77E + 00
77-47-4	Hexachlorocyclopentadiene	1.63E + 04	1.10E + 04	6.65E + 03
78-59-1	Isophorone	2.96E + 05	2.91E + 05	1.42E + 05
78-87-5	Propylene Dichloride	5.26E + 04	3.41E + 04	3.26E + 04
79-00-5	1,1,2-Trichloroethane	4.35E + 04	3.67E + 04	6.89E + 04
79-06-1	Acrylamide	1.46E + 04	9.38E + 03	1.75E + 04
79-10-7	Acrylic Acid	1.31E + 05	1.32E + 05	1.54E + 05
79-46-9	2-Nitropropane	2.79E + 04	1.55E + 04	1.46E + 04
80-62-6	Methyl Methacrylate	9.90E + 05	1.42E + 06	1.16E + 06
82-68-8	Pentachloronitrobenzene	2.07E + 03	6.60E + 01	6.72E + 01
83-32-9	Acenaphthene	2.11E + 05	2.36E + 05	2.41E + 05
84-74-2	Dibutyl Phthalate	5.97E + 04	6.30E + 04	5.99E + 04
85-44-9	Phthalic Anhydride	8.70E + 04	1.05E + 05	1.16E + 05
86-73-7	Fluorene	4.50E + 05	4.60E + 05	5.33E + 05
87-86-5	Pentachlorophenol	2.96E + 03	2.67E + 03	2.07E + 03
91-20-3	Naphthalene	1.09E + 07	1.57E + 07	1.35E + 07
91-57-6	2-Methylnaphthalene	5.41E + 04	5.10E + 05	2.51E + 05
92-52-4	Biphenyl	2.35E + 05	2.79E + 05	2.21E + 05
92-87-5	Benzidine	1.38E + 02	8.72E + 02	7.12E + 03
94-75-7	2,4-Dichlorophenoxyacetic Acid	1.27E + 07	4.04E + 02	7.90E + 06
95-48-7	o-Cresol	3.88E + 04	2.64E + 04	3.83E + 04
95-95-4	2,4,5-Trichlorophenol	7.22E + 03	7.44E + 03	9.58E + 03
96-12-8	1,2-Dibromo-3-Chloropropane	2.04E + 02	2.25E + 02	1.66E + 02
96-45-7	Ethylene Thiourea	2.85E + 02	6.06E + 02	1.67E + 02
98-82-8	Cumene	9.13E + 05	8.37E + 05	9.16E + 05
98-86-2	Acetophenone	1.49E + 05	1.63E + 05	1.84E + 05
98-95-3	Nitrobenzene	2.74E + 04	1.24E + 04	1.16E + 04

a. From 2011, 2014, and 2017 National Emission Inventory.

Table S2. Chemical analyzed and relevant data used in this study.

0	Name	Total emission (kg)[a]	ED50,inhalation ($mg_{chemical}/kg_{body-weight}/d$)[b]	ED50,ingestion ($mg_{chemical}/kg_{body-weight}/d$)[b]	Dissociation, pKa[c]	Dissociation, pKb[c]	log K_{OW}[c]	log K_{OA}[c]	log $k_{(OH)}$[c]	Half-life of biodegradation in the environment (day)[c, d]	Half-life of biotransformation in fish (day)[c]	Half-life of biotransformation in human (day)[c]
100-41-4	Ethyl Benzene	5.36E + 07	7.25E + 02	1.07E + 02	NaN	NaN	3.15E + 00	3.71E + 00	−1.12E + 01	1.14E + 01	−2.18E − 01	6.77E − 01
100-42-5	Styrene	2.14E + 07	2.84E + 01	6.12E + 02	NaN	NaN	2.95E + 00	3.84E + 00	−1.02E + 01	1.07E + 01	−3.17E − 01	−7.60E − 01
106-46-7	1,4-Dichlorobenzene	2.84E + 05	1.25E + 02	1.25E + 02	NaN	NaN	3.44E + 00	4.44E + 00	−1.25E + 01	3.20E + 01	8.61E − 01	1.96E − 01
106-88-7	1,2-Epoxybutane	9.40E + 03	2.00E + 00	2.00E + 00	NaN	NaN	6.80E − 01	2.76E + 00	−1.17E + 01	1.35E + 01	−6.72E − 01	−4.19E − 01
106-89-8	Epichlorohydrin	5.60E + 04	6.02E − 01	6.02E − 01	NaN	NaN	4.50E − 01	3.09E + 00	−1.24E + 01	1.85E + 01	−9.46E − 01	−6.95E − 02
106-93-4	Ethylene Dibromide	1.99E + 04	5.94E + 01	5.94E + 01	NaN	NaN	1.96E + 00	3.60E + 00	−1.26E + 01	2.17E + 01	−6.12E − 01	−1.56E − 01
106-99-0	1,3-Butadiene	1.51E + 07	3.31E + 00	3.31E + 00	NaN	NaN	1.99E + 00	1.76E + 00	−1.02E + 01	9.79E + 00	−6.87E − 01	−9.06E − 01
107-02-8	Acrolein	1.01E + 07	4.68E − 03	1.10E − 01	NaN	NaN	−1.00E−02	2.20E + 00	−1.07E + 01	7.44E + 00	−6.55E − 01	−9.06E − 01
107-05-1	Allyl Chloride	1.28E + 04	3.01E + 00	3.01E + 00	NaN	NaN	1.55E + 00	2.08E + 00	−1.08E + 01	1.47E + 01	−6.83E − 01	−4.73E − 01
107-13-1	Acrylonitrile	2.65E + 05	7.93E − 01	7.93E − 01	NaN	NaN	2.50E − 01	2.47E + 00	−1.10E + 01	1.19E + 01	−6.63E − 01	−6.82E − 01
107-21-1	Ethylene Glycol	4.11E + 07	4.40E + 02	4.40E + 02	1.10E + 01	NaN	−1.36E + 00	5.45E + 00	−1.08E + 01	8.00E + 00	−1.39E + 00	−1.11E + 00
108-05-4	Vinyl Acetate	5.95E + 05	8.35E + 00	8.35E + 00	NaN	NaN	7.30E − 01	2.58E + 00	−1.06E + 01	8.06E + 00	−8.76E − 01	−1.25E + 00
108-10-1	Methyl Isobutyl Ketone	8.03E + 06	1.71E + 03	1.71E + 03	NaN	NaN	1.31E + 00	3.57E + 00	−1.09E + 01	1.17E + 01	−7.67E − 01	−1.24E + 00
108-31-6	Maleic Anhydride	1.19E + 05	2.20E + 01	2.20E + 01	NaN	NaN	5.98E − 01	4.95E + 00	−1.18E + 01	1.71E + 01	−7.00E − 01	−1.30E + 00
108-39-4	m-Cresol	9.87E + 03	5.50E + 01	5.50E + 01	1.02E + 01	NaN	1.96E + 00	5.73E + 00	−1.02E + 01	1.53E + 01	−8.07E − 01	−1.22E + 00
108-88-3	Toluene	3.48E + 08	7.68E + 01	2.45E + 02	NaN	NaN	2.73E + 00	3.40E + 00	−1.12E + 01	1.80E + 01	−4.96E − 01	−8.07E − 01
108-90-7	Chlorobenzene	4.31E + 05	5.81E + 01	5.81E + 01	NaN	NaN	2.84E + 00	3.56E + 00	−1.21E + 01	1.82E + 01	−4.90E − 01	−3.27E − 01
108-95-2	Phenol	9.73E + 06	8.49E + 01	8.49E + 01	1.00E + 01	NaN	1.46E + 00	5.56E + 00	−1.06E + 01	5.00E + 00	−9.02E − 01	5.67E − 01
109-86-4	Ethylene Glycol Methyl E	1.79E + 05	1.42E + 01	1.42E + 01	1.16E + 01	NaN	−7.70E − 01	4.03E + 00	−1.09E + 01	1.15E + 01	−1.27E + 00	−1.06E + 00
110-54-3	Hexane	9.19E + 07	3.05E + 01	3.05E + 01	NaN	NaN	3.90E + 00	2.30E + 00	−1.13E + 01	7.48E + 00	−2.13E − 01	−7.40E − 01
110-80-5	Cellosolve Solvent	2.99E + 03	5.68E + 01	5.68E + 01	1.03E + 01	NaN	−3.20E − 01	4.32E + 00	−1.08E + 01	1.19E + 01	−1.22E + 00	−9.25E − 01
114-26-1	Propoxur	1.60E − 01	1.80E + 00	1.80E + 00	6.81E + 00	NaN	1.52E + 00	8.82E + 00	−1.07E + 01	2.18E + 01	−1.02E + 00	−1.48E + 00
117-81-7	Bis(2-Ethylhexyl)Phthala	1.95E + 05	6.87E + 00	6.87E + 00	NaN	NaN	7.60E + 00	1.21E + 01	−1.07E + 01	4.80E + 00	5.15E − 01	−1.29E − 01
118-74-1	Hexachlorobenzene	1.24E + 03	2.75E + 00	2.75E + 00	NaN	NaN	5.73E + 00	7.36E + 00	−1.36E + 01	5.84E + 02	1.77E + 00	4.39E + 00
120-12-7	Anthracene	1.75E + 05	6.18E + 02	6.18E + 02	NaN	NaN	4.45E + 00	7.32E + 00	−9.75E + 00	5.47E + 01	1.46E − 01	−6.75E − 01
120-82-1	1,2,4-Trichlorobenzene	1.01E + 05	3.25E + 01	3.25E + 01	NaN	NaN	4.02E + 00	5.11E + 00	−1.23E + 01	6.10E + 01	1.06E + 00	7.33E − 02
121-14-2	2,4-Dinitrotoluene	6.15E + 03	1.22E + 00	1.22E + 00	NaN	NaN	1.98E + 00	6.36E + 00	−1.22E + 01	1.04E + 02	−4.19E − 01	3.03E − 01
121-44-8	Triethylamine	1.70E + 05	1.63E + 01	1.63E + 01	NaN	1.06E + 01	1.45E + 00	3.42E + 00	−1.03E + 01	2.90E + 01	−2.32E − 01	−5.55E − 01
121-69-7	N,N-Dimethylaniline	6.81E + 03	3.45E + 00	3.45E + 00	NaN	5.30E + 00	2.31E + 00	4.69E + 00	−9.83E + 00	2.57E + 01	−8.75E − 01	−7.53E − 01
123-31-9	Hydroquinone	3.89E + 04	1.32E + 01	1.32E + 01	9.92E + 00	NaN	5.90E − 01	8.45E + 00	−1.06E + 01	1.20E + 01	−9.61E − 01	−8.72E − 01
127-18-4	Tetrachloroethylene	7.53E + 06	8.65E + 00	8.65E + 00	NaN	NaN	3.40E + 00	3.53E + 00	−1.28E + 01	5.16E + 01	−3.88E − 01	1.13E + 00
129-00-0	Pyrene	4.72E + 05	4.64E + 01	4.64E + 01	NaN	NaN	4.88E + 00	8.52E + 00	−1.03E + 01	1.66E + 02	3.56E − 01	−4.59E − 01

133-06-2	Captan	2.27E + 05	2.75E + 01	2.75E + 01	NaN	2.07E + 00	2.80E + 00	9.09E + 00	−1.03E + 01	1.53E + 02	−1.86E − 02	5.05E − 01
133-90-4	Chloramben	2.00E − 02	4.64E + 00	4.64E + 00	3.12E + 00	NaN	1.41E + 00	9.39E + 00	−1.12E + 01	6.29E + 01	−1.03E + 00	1.37E − 01
1582-09-8	Trifluralin	2.81E + 06	4.59E + 00	4.59E + 00	NaN	2.84E + 00	5.34E + 00	8.07E + 00	−1.07E + 01	6.61E + 02	8.65E − 02	−7.39E − 01
1634-04-4	Methyl Tert-Butyl Ether	2.10E + 05	4.33E + 02	4.33E + 02	NaN	NaN	9.40E − 01	2.62E + 00	−1.15E + 01	3.07E + 01	−6.96E − 01	−8.93E − 01
206-44-0	Fluoranthene	2.84E + 05	7.73E + 01	7.73E + 01	NaN	NaN	5.05E + 00	8.85E + 00	−1.02E + 01	3.06E + 02	−1.88E−02	−8.93E − 01
50-00-0	Formaldehyde	6.56E + 08	3.30E + 01	3.30E + 01	NaN	NaN	3.50E − 01	3.21E + 00	−1.11E + 01	6.47E + 00	−7.62E − 01	−1.08E + 00
510-15-6	Chlorobenzilate	2.18E + 01	4.40E + 00	4.40E + 00	8.31E + 00	NaN	4.74E + 00	1.05E + 01	−1.11E + 01	9.60E + 01	6.45E − 01	3.21E − 01
51-28-5	2,4-Dinitrophenol	6.04E + 03	4.50E + 00	4.50E + 00	4.85E + 00	NaN	1.67E + 00	7.23E + 00	−1.21E + 01	7.28E + 01	−9.91E − 01	−1.05E + 00
532-27-4	2-Chloroacetophenone	6.65E + 02	1.25E − 02	1.25E − 02	NaN	NaN	1.84E + 00	5.38E + 00	−1.17E + 01	1.77E + 01	−5.33E − 01	−6.23E − 01
542-75-6	1,3-Dichloropropene	1.91E + 05	2.67E − 02	4.11E + 00	NaN	NaN	2.03E + 00	2.98E + 00	−1.10E + 01	2.10E + 01	−4.85E − 01	6.12E − 02
56-23-5	Carbon Tetrachloride	1.70E + 05	7.81E − 01	7.81E − 01	NaN	NaN	2.83E + 00	2.78E + 00	−1.59E + 01	1.04E + 02	−5.43E − 01	1.03E + 00
56-38-2	Parathion	1.34E + 01	8.99E − 01	8.99E − 01	6.60E + 00	NaN	3.83E + 00	9.04E + 00	−1.04E + 01	3.55E + 01	−1.81E − 01	5.79E − 02
57-74-9	Chlordane	5.56E + 00	5.43E − 01	1.10E − 01	NaN	NaN	6.22E + 00	8.96E + 00	−1.17E + 01	2.14E + 03	2.04E + 00	3.32E + 00
58-89-9	1,2,3,4,5,6-Hexachlorocy	2.83E + 01	3.63E − 01	3.63E − 01	NaN	NaN	4.14E + 00	7.95E + 00	−1.27E + 01	1.83E + 02	1.51E + 00	3.30E + 00
593-60-2	Vinyl Bromide	4.64E + 01	3.22E + 00	3.22E + 00	NaN	NaN	1.57E + 00	2.16E + 00	−1.12E + 01	1.35E + 01	−5.63E − 01	−5.31E − 01
62-53-3	Aniline	6.45E + 04	2.84E + 00	2.84E + 00	NaN	4.50E + 00	9.00E − 01	4.96E + 00	−9.95E + 00	2.39E + 01	−9.50E − 01	−8.62E − 01
62-73-7	Dichlorvos	2.51E + 01	8.35E − 02	3.60E − 01	NaN	NaN	1.43E + 00	6.07E + 00	−1.05E + 01	2.43E + 01	−1.72E − 01	5.99E − 01
63-25-2	Carbaryl	3.53E + 05	4.64E + 00	4.64E + 00	8.14E + 00	NaN	2.36E + 00	9.18E + 00	−1.07E + 01	2.64E + 01	−1.05E + 00	−8.41E − 01
67-56-1	Methanol	2.03E + 09	5.50E + 02	5.50E + 02	1.03E + 01	NaN	−7.70E − 01	2.91E + 00	−1.20E + 01	7.42E + 00	−1.35E + 00	−1.06E + 00
67-66-3	Chloroform	1.01E + 06	8.61E + 00	8.61E + 00	NaN	NaN	1.97E + 00	2.77E + 00	−1.30E + 01	3.94E + 01	−3.73E − 01	2.19E − 01
67-72-1	Hexachloroethane	3.36E + 04	1.10E + 00	1.10E + 00	NaN	NaN	4.14E + 00	4.70E + 00	−1.43E + 01	4.31E + 02	6.76E − 01	2.10E + 00
68-12-2	N,N-Dimethylformamide	1.02E + 05	3.30E + 00	3.30E + 00	NaN	4.04E + 00	−1.01E + 00	4.33E + 00	−1.09E + 01	9.92E + 00	−9.87E − 01	−9.00E − 01
71-43-2	Benzene	1.21E + 08	7.52E + 01	7.52E + 01	NaN	NaN	2.13E + 00	2.80E + 00	−1.19E + 01	4.00E + 01	−8.16E − 01	−3.31E − 01
71-55-6	Methyl Chloroform	9.80E + 06	2.59E + 03	2.59E + 03	NaN	NaN	2.49E + 00	2.69E + 00	−1.40E + 01	4.27E + 01	−6.81E − 01	5.48E − 01
72-43-5	Methoxychlor	6.56E + 00	2.20E + 01	2.20E + 01	NaN	NaN	5.08E + 00	9.67E + 00	−1.05E + 01	1.91E + 02	1.17E + 00	1.08E + 00
74-83-9	Methyl Bromide	9.42E + 05	1.85E − 01	8.09E + 01	NaN	NaN	1.19E + 00	1.86E + 00	−1.35E + 01	1.54E + 01	−6.58E − 01	−5.79E − 01
74-87-3	Methyl Chloride	1.26E + 06	3.16E + 01	3.16E + 01	NaN	NaN	9.10E − 01	1.38E + 00	−1.34E + 01	1.68E + 01	−6.03E − 01	−5.20E − 01
75-00-3	Ethyl Chloride	2.38E + 05	6.68E + 03	6.68E + 03	NaN	NaN	1.43E + 00	1.98E + 00	−1.24E + 01	1.43E + 01	−6.84E − 01	−3.90E − 01
75-01-4	Vinyl Chloride	3.87E + 05	4.18E + 00	3.48E − 01	NaN	NaN	1.46E + 00	1.68E + 00	−1.12E + 01	7.60E + 01	−5.04E − 01	−4.73E − 01
75-05-8	Acetonitrile	7.19E + 05	1.00E + 02	1.00E + 02	NaN	NaN	−3.40E − 01	2.40E + 00	−1.36E + 01	1.06E + 01	−7.24E − 01	−7.30E − 01
75-07-0	Acetaldehyde	4.47E + 08	7.27E + 00	7.27E + 00	NaN	NaN	−3.40E − 01	2.01E + 00	−1.08E + 01	6.62E + 00	−7.09E − 01	−9.54E − 01
75-09-2	Methylene Chloride	3.15E + 06	1.29E + 01	1.29E + 01	NaN	NaN	1.25E + 00	2.21E + 00	−1.30E + 01	2.58E + 01	−6.76E − 01	−4.02E − 02
75-15-0	Carbon Disulfide	4.13E + 06	1.06E + 00	4.07E + 01	NaN	NaN	1.94E + 00	2.25E + 00	−1.15E + 01	1.52E + 01	−6.66E − 01	−4.97E − 01
75-25-2	Bromoform	1.60E + 04	1.97E + 01	1.97E + 01	NaN	NaN	2.40E + 00	4.02E + 00	−1.28E + 01	3.37E + 01	−5.16E − 01	3.20E − 01
75-35-4	Vinylidene Chloride	1.70E + 04	2.96E + 01	7.04E + 00	NaN	NaN	2.13E + 00	2.35E + 00	−1.10E + 01	2.20E + 01	−5.53E − 01	6.12E − 02
75-56-9	Propylene Oxide	2.49E + 05	1.21E + 00	1.21E + 00	NaN	NaN	3.00E − 02	2.39E + 00	−1.23E + 01	1.31E + 01	−8.35E − 01	−5.50E − 01
76-44-8	Heptachlor	2.77E + 00	2.86E + 00	2.86E + 00	NaN	NaN	5.47E + 00	7.58E + 00	−1.02E + 01	1.72E + 03	1.70E + 00	2.63E + 00
77-47-4	Hexachlorocyclopentadiene	6.65E + 03	4.01E − 02	3.40E + 00	NaN	NaN	5.04E + 00	5.95E + 00	−1.24E + 01	3.00E + 02	8.58E − 01	2.10E + 00

78-59-1	Isophorone	1.42E + 05	4.59E + 02	4.59E + 02	NaN	NaN	1.70E + 00	4.79E + 00	−1.06E + 01	1.91E + 01	−8.20E − 01	−7.17E − 01
78-87-5	Propylene Dichloride	3.26E + 04	2.71E − 01	2.71E − 01	NaN	NaN	1.98E + 00	2.95E + 00	−1.23E + 01	2.29E + 01	−6.48E − 01	1.39E − 02
79-00-5	1,1,2-Trichloroethane	6.89E + 04	2.41E + 00	2.41E + 00	NaN	NaN	1.89E + 00	3.36E + 00	−1.27E + 01	2.84E + 01	−5.83E − 01	4.94E − 01
79-06-1	Acrylamide	1.75E + 04	2.20E − 01	2.20E − 01	9.26E + 00	NaN	−6.70E − 01	5.39E + 00	−1.08E + 01	1.09E + 01	−1.03E + 00	−1.30E + 00
79-10-7	Acrylic Acid	1.54E + 05	6.89E − 02	1.17E + 02	4.15E + 00	NaN	3.50E − 01	4.50E + 00	−1.08E + 01	5.94E + 00	−1.07E + 00	−1.30E + 00
79-46-9	2-Nitropropane	1.46E + 04	6.68E + 00	6.68E + 00	NaN	NaN	9.30E − 01	2.75E + 00	−1.26E + 01	1.59E + 01	−7.93E − 01	−1.55E + 00
80-62-6	Methyl Methacrylate	1.16E + 06	3.43E + 00	2.99E + 02	NaN	NaN	1.38E + 00	3.20E + 00	−1.04E + 01	8.19E + 00	−1.05E + 00	−1.20E + 00
82-68-8	Pentachloronitrobenzene	6.72E + 01	2.20E + 00	2.20E + 00	NaN	NaN	4.64E + 00	7.47E + 00	−1.34E + 01	1.46E + 02	3.39E − 01	1.22E + 00
83-32-9	Acenaphthene	2.41E + 05	1.08E + 02	1.08E + 02	NaN	NaN	3.92E + 00	6.19E + 00	−1.00E + 01	2.45E + 01	1.64E − 01	−6.56E − 01
84-74-2	Dibutyl Phthalate	5.99E + 04	1.37E + 02	1.37E + 02	NaN	NaN	4.50E + 00	8.74E + 00	−1.09E + 01	3.02E + 00	−4.00E − 01	−1.10E + 00
85-44-9	Phthalic Anhydride	1.16E + 05	4.83E + 02	4.83E + 02	NaN	NaN	1.60E + 00	6.86E + 00	−1.13E + 01	2.12E + 01	−9.31E − 01	−1.07E + 00
86-73-7	Fluorene	5.33E + 05	7.73E + 01	7.73E + 01	NaN	NaN	4.18E + 00	6.71E + 00	−1.09E + 01	2.45E + 01	6.48E − 01	−1.20E − 01
87-86-5	Pentachlorophenol	2.07E + 03	6.60E + 00	6.60E + 00	6.95E + 00	NaN	5.12E + 00	9.79E + 00	−1.25E + 01	2.82E + 02	2.64E − 03	1.50E + 00
91-20-3	Naphthalene	1.35E + 07	3.88E + 00	2.17E + 01	NaN	NaN	3.30E + 00	5.11E + 00	−1.06E + 01	1.51E + 01	4.09E − 01	−8.93E − 01
91-57-6	2-Methylnaphthalene	2.51E + 05	1.67E + 01	1.67E + 01	NaN	NaN	3.86E + 00	5.68E + 00	−1.03E + 01	1.74E + 01	3.07E − 01	−1.05E + 00
92-52-4	Biphenyl	2.21E + 05	5.50E + 01	5.50E + 01	NaN	NaN	4.01E + 00	6.03E + 00	−1.11E + 01	2.10E + 01	6.21E − 01	2.78E − 01
92-87-5	Benzidine	7.12E + 03	8.35E − 01	8.35E − 01	NaN	4.79E + 00	1.34E + 00	9.18E + 00	−1.01E + 01	6.65E + 01	−6.72E − 01	−8.72E − 01
94-75-7	2,4-Dichlorophenoxy Acet	7.90E + 06	6.12E + 00	6.12E + 00	2.42E + 00	NaN	2.81E + 00	8.94E + 00	−1.11E + 01	2.22E + 01	−5.42E − 01	−8.49E − 01
95-48-7	o-Cresol	3.83E + 04	5.50E + 01	5.50E + 01	1.01E + 01	NaN	1.95E + 00	5.65E + 00	−1.04E + 01	1.53E + 01	−7.49E − 01	−1.59E + 00
95-95-4	2,4,5-Trichlorophenol	9.58E + 03	1.10E + 02	1.10E + 02	7.15E + 00	NaN	3.72E + 00	7.63E + 00	−1.15E + 01	8.38E + 01	−2.40E − 01	3.76E − 01
96-12-8	1,2-Dibromo-3-Chloroprop	1.66E + 02	1.42E − 01	1.42E − 01	NaN	NaN	2.96E + 00	4.81E + 00	−1.24E + 01	2.81E + 01	−7.08E − 01	3.78E − 01
96-45-7	Ethylene Thiourea	1.67E + 02	2.39E + 00	2.39E + 00	NaN	4.59E + 00	−6.60E − 01	8.49E + 00	−1.03E + 01	1.06E + 01	−9.13E − 01	−2.54E − 01
98-82-8	Cumene	9.16E + 05	3.63E + 02	1.21E + 02	NaN	NaN	3.66E + 00	3.99E + 00	−1.12E + 01	1.64E + 01	−2.41E − 01	−7.53E − 01
98-86-2	Acetophenone	1.84E + 05	4.65E + 02	4.65E + 02	NaN	NaN	1.58E + 00	4.69E + 00	−1.16E + 01	6.00E + 00	−6.63E − 01	−1.10E + 00
98-95-3	Nitrobenzene	1.16E + 04	1.26E + 00	1.26E + 00	NaN	NaN	1.85E + 00	4.36E + 00	−1.29E + 01	3.17E + 01	−7.31E − 01	2.07E + 00

Table S3. Comparison between PROTEX predicted air concentration (μg/m3) and predictions from U.S. EPA's National Air Toxics Assessment (NATA).

CASRN	Chemical name	PROTEX prediction	NATA prediction[a]		
			mean	2.5th pctl	97.5th pctl
107-02-8	Acrolein	8.09E − 03	2.61E − 02	5.75E − 03	6.71E − 02
127-18-4	Tetrachloroethylene	1.18E − 01	2.09E − 02	1.19E − 02	8.29E − 02
108-88-3	Toluene	1.24E + 00	8.57E − 01	9.84E − 02	3.49E + 00
542-75-6	1,3-Dichloropropene	3.63E − 04	3.38E − 05	0	1.93E − 02
71-43-2	Benzene	1.19E + 00	3.91E − 01	1.28E − 01	9.45E − 01
50-00-0	Formaldehyde	6.33E − 01	1.34E + 00	6.59E − 01	2.24E + 00
77-47-4	Hexachlorocyclopentadien	9.06E − 05	7.25E − 09	0	2.29E − 06
91-20-3	Naphthalene	1.03E − 02	3.18E − 02	5.45E − 03	1.04E − 01
56-23-5	Carbon Tetrachloride	2.89E − 03	5.34E − 01	4.56E − 01	5.52E − 01
75-07-0	Acetaldehyde	4.38E − 01	1.03E + 00	5.23E − 01	2.09E + 00

a. Concentrations are summarized from the 2014 National Air Toxics Assessment (https://www.epa.gov/national-air-toxics-assessment/2014-nata-assessment-results; accessed on July 08, 2021).

Table S4. Exposure as a fraction to ED50 and estimated risk for the 95 organic chemicals analyzed.

CASRN	Chemical name	Average daily dose/ED_{50} ratio			PrHE[a]		
		3-year-old	14-year-old	25-year-old	3-year-old	14-year-old	25-year-old
100-41-4	Ethyl Benzene	8.05E − 06	2.80E − 06	2.57E − 06	8.85E − 86	1.68E − 101	7.74E − 103
100-42-5	Styrene	5.42E − 06	1.88E − 06	1.73E − 06	1.64E − 91	8.77E − 108	4.05E − 109
106-46-7	1,4-Dichlorobenzene	1.12E − 06	3.91E − 07	3.59E − 07	3.09E − 116	2.06E − 134	6.02E − 136
106-88-7	1,2-Epoxybutane	3.45E − 07	1.19E − 07	1.06E − 07	1.15E − 136	1.43E − 156	8.09E − 159
106-89-8	Epichlorohydrin	4.48E − 06	1.54E − 06	1.35E − 06	2.44E − 94	5.31E − 111	3.76E − 113
106-93-4	Ethylene Dibromide	1.25E − 07	4.33E − 08	3.97E − 08	1.27E − 155	9.47E − 177	1.54E − 178
106-99-0	1,3-Butadiene	1.06E − 07	3.67E − 08	3.37E − 08	8.09E − 159	3.62E − 180	6.06E − 182
107-02-8	Acrolein	5.47E − 03	1.86E − 03	1.55E − 03	1.66E − 18	4.23E − 26	1.60E − 27
107-05-1	Allyl Chloride	3.78E − 09	1.31E − 09	1.20E − 09	1.65E − 230	4.09E − 256	2.70E − 258
107-13-1	Acrylonitrile	3.50E − 06	1.20E − 06	1.03E − 06	4.56E − 98	4.31E − 115	1.23E − 117
107-21-1	Ethylene Glycol	1.71E − 09	5.66E − 10	4.32E − 10	1.51E − 249	2.34E − 277	2.24E − 284
108-05-4	Vinyl Acetate	6.37E − 07	2.20E − 07	1.97E − 07	8.12E − 126	6.53E − 145	5.66E − 147
108-10-1	Methyl Isobutyl Ketone	1.50E − 07	5.21E − 08	4.75E − 08	3.99E − 152	5.78E − 173	7.51E − 175
108-31-6	Maleic Anhydride	1.06E − 07	3.67E − 08	3.25E − 08	8.09E − 159	3.62E − 180	1.06E − 182
108-39-4	m-Cresol	5.44E − 10	1.89E − 10	1.73E − 10	2.21E − 278	2.09E − 306	0.00E + 00
108-88-3	Toluene	5.45E − 04	1.89E − 04	1.74E − 04	1.91E − 36	8.34E − 47	1.05E − 47
108-90-7	Chlorobenzene	2.97E − 06	1.03E − 06	9.46E − 07	1.37E − 100	1.23E − 117	4.61E − 119
108-95-2	Phenol	3.99E − 07	1.38E − 07	1.26E − 07	4.74E − 134	1.02E − 153	1.81E − 155
109-86-4	Ethylene Glycol Methyl Ether	1.61E − 09	5.44E − 10	4.55E − 10	5.03E − 251	2.21E − 278	5.06E − 283
110-54-3	Hexane	1.68E − 05	5.83E − 06	5.35E − 06	1.27E − 75	1.93E − 90	1.06E − 91
110-80-5	Cellosolve Solvent	1.26E − 11	4.28E − 12	3.56E − 12	0.00E + 00	0.00E + 00	0.00E + 00
114-26-1	Propoxur	6.01E − 13	2.08E − 13	1.86E − 13	0.00E + 00	0.00E + 00	0.00E + 00
117-81-7	Bis(2-Ethylhexyl)Phthalate	1.69E − 06	5.88E − 07	5.40E − 07	1.70E − 109	3.31E − 127	1.08E − 128
118-74-1	Hexachlorobenzene	6.83E − 07	2.37E − 07	2.18E − 07	1.30E − 124	1.57E − 143	4.41E − 145
120-12-7	Anthracene	3.50E − 10	1.21E − 10	1.12E − 10	6.59E − 290	1.24E − 318	8.88E − 321
120-82-1	1,2,4-Trichlorobenzene	1.61E − 06	5.61E − 07	5.15E − 07	2.80E − 110	5.01E − 128	1.59E − 129
121-14-2	2,4-Dinitrotoluene	4.43E − 07	1.54E − 07	1.41E − 07	3.47E − 132	1.27E − 151	2.62E − 153
121-44-8	Triethylamine	1.13E − 08	3.92E − 09	3.54E − 09	1.78E − 205	1.18E − 229	4.71E − 232
121-69-7	N,N-Dimethylaniline	2.65E − 09	9.22E − 10	8.46E − 10	6.13E − 239	6.68E − 265	4.47E − 267
123-31-9	Hydroquinone	8.79E − 10	3.03E − 10	2.68E − 10	4.16E − 266	9.97E − 294	5.34E − 297
127-18-4	Tetrachloroethylene	5.96E − 04	2.07E − 04	1.90E − 04	1.25E − 35	7.08E − 46	9.20E − 47
129-00-0	Pyrene	1.03E − 07	3.56E − 08	3.27E − 08	2.23E − 159	8.42E − 181	1.42E − 182
133-06-2	Captan	2.40E − 07	8.33E − 08	7.65E − 08	2.69E − 143	1.50E − 163	3.08E − 165
133-90-4	Chloramben	1.47E − 15	4.82E − 16	3.59E − 16	0.00E + 00	0.00E + 00	0.00E + 00
1582-09-8	Trifluralin	2.76E − 05	9.57E − 06	8.79E − 06	3.87E − 69	2.48E − 83	1.57E − 84
1634-04-4	Methyl Tert-Butyl Ether	5.85E − 08	2.03E − 08	1.84E − 08	1.29E − 170	1.11E − 192	8.49E − 195

206-44-0	Fluoranthene	2.09E − 08	7.26E − 09	6.66E − 09	4.70E − 192	2.05E − 215	2.22E − 217
50-00-0	Formaldehyde	1.72E − 04	5.89E − 05	5.11E − 05	7.75E − 48	8.22E − 60	1.66E − 61
510-15-6	Chlorobenzilate	1.12E − 10	3.90E − 11	3.58E − 11	8.88E − 321	0.00E + 00	0.00E + 00
51-28-5	2,4-Dinitrophenol	5.84E − 09	1.94E − 09	1.49E − 09	2.17E − 220	1.81E − 246	6.25E − 253
532-27-4	2-Chloroacetophenone	3.33E − 06	1.16E − 06	1.06E − 06	7.90E − 99	1.18E − 115	3.73E − 117
542-75-6	1,3-Dichloropropene	3.89E − 04	1.35E − 04	1.24E − 04	1.33E − 39	2.16E − 50	2.43E − 51
56-23-5	Carbon Tetrachloride	8.69E − 05	3.02E − 05	2.77E − 05	2.70E − 55	5.40E − 68	4.31E − 69
56-38-2	Parathion	1.19E − 10	4.14E − 11	3.80E − 11	4.27E − 319	0.00E + 00	0.00E + 00
57-74-9	Chlordane	8.58E − 09	2.98E − 09	2.74E − 09	1.24E − 211	3.89E − 236	3.86E − 238
58-89-9	1,2,3,4,5,6-Hexachlorocy	2.66E − 08	9.26E − 09	8.50E − 09	6.46E − 187	6.42E − 210	7.62E − 212
593-60-2	Vinyl Bromide	5.87E − 11	2.04E − 11	1.86E − 11	0.00E + 00	0.00E + 00	0.00E + 00
62-53-3	Aniline	5.23E − 09	1.81E − 09	1.63E − 09	6.19E − 223	3.70E − 248	1.01E − 250
62-73-7	Dichlorvos	2.86E − 09	9.92E − 10	9.05E − 10	4.08E − 237	4.66E − 263	2.27E − 265
63-25-2	Carbaryl	5.33E − 07	1.85E − 07	1.70E − 07	6.36E − 129	3.74E − 148	9.51E − 150
67-56-1	Methanol	9.77E − 07	3.36E − 07	2.97E − 07	1.61E − 118	3.84E − 137	2.20E − 139
67-66-3	Chloroform	4.08E − 05	1.42E − 05	1.30E − 05	3.05E − 64	6.93E − 78	4.35E − 79
67-72-1	Hexachloroethane	3.19E − 05	1.11E − 05	1.02E − 05	2.65E − 67	2.90E − 81	1.93E − 82
68-12-2	N,N-Dimethylformamide	4.77E − 09	1.62E − 09	1.38E − 09	4.54E − 225	7.14E − 251	7.98E − 255
71-43-2	Benzene	3.67E − 04	1.27E − 04	1.17E − 04	3.61E − 40	4.93E − 51	5.52E − 52
71-55-6	Methyl Chloroform	1.07E − 06	3.73E − 07	3.42E − 07	5.35E − 117	2.94E − 135	8.03E − 137
72-43-5	Methoxychlor	5.70E − 12	1.98E − 12	1.82E − 12	0.00E + 00	0.00E + 00	0.00E + 00
74-83-9	Methyl Bromide	2.03E − 05	7.05E − 06	6.40E − 06	4.08E − 73	1.11E − 87	4.43E − 89
74-87-3	Methyl Chloride	1.61E − 07	5.60E − 08	5.13E − 08	8.86E − 151	1.69E − 171	2.80E − 173
75-00-3	Ethyl Chloride	3.07E − 10	1.07E − 10	9.75E − 11	2.22E − 293	4.74E − 322	0.00E + 00
75-01-4	Vinyl Chloride	8.05E − 07	2.80E − 07	2.57E − 07	8.51E − 122	1.85E − 140	4.92E − 142
75-05-8	Acetonitrile	3.80E − 08	1.28E − 08	1.05E − 08	1.91E − 179	1.02E − 202	4.15E − 207
75-07-0	Acetaldehyde	8.40E − 05	2.84E − 05	2.33E − 05	1.11E − 55	8.96E − 69	2.57E − 71
75-09-2	Methylene Chloride	9.75E − 06	3.38E − 06	3.08E − 06	4.52E − 83	1.34E − 98	4.99E − 100
75-15-0	Carbon Disulfide	3.59E − 05	1.25E − 05	1.14E − 05	7.98E − 66	1.26E − 79	6.80E − 81
75-25-2	Bromoform	3.74E − 07	1.30E − 07	1.19E − 07	3.28E − 135	7.25E − 155	1.43E − 156
75-35-4	Vinylidene Chloride	3.86E − 09	1.34E − 09	1.23E − 09	5.14E − 230	1.49E − 255	1.11E − 257
75-56-9	Propylene Oxide	4.62E − 06	1.57E − 06	1.33E − 06	7.05E − 94	1.09E − 110	2.14E − 113
76-44-8	Heptachlor	1.49E − 11	5.19E − 12	4.76E − 12	0.00E + 00	0.00E + 00	0.00E + 00
77-47-4	Hexachlorocyclopentadiene	1.38E − 04	4.79E − 05	4.39E − 05	3.47E − 50	2.76E − 62	2.40E − 63
78-59-1	Isophorone	5.16E − 09	1.79E − 09	1.64E − 09	3.02E − 223	1.98E − 248	1.43E − 250
78-87-5	Propylene Dichloride	4.34E − 05	1.51E − 05	1.38E − 05	1.74E − 63	4.70E − 77	2.83E − 78
79-00-5	1,1,2-Trichloroethane	1.20E − 05	4.16E − 06	3.81E − 06	3.47E − 80	1.89E − 95	8.90E − 97
79-06-1	Acrylamide	1.69E − 08	5.65E − 09	4.51E − 09	1.22E − 196	3.76E − 221	2.25E − 226
79-10-7	Acrylic Acid	3.21E − 08	1.05E − 08	7.84E − 09	5.83E − 183	4.15E − 207	1.14E − 213
79-46-9	2-Nitropropane	3.90E − 07	1.35E − 07	1.22E − 07	1.85E − 134	3.85E − 154	4.32E − 156
80-62-6	Methyl Methacrylate	2.81E − 06	9.74E − 07	8.88E − 07	1.90E − 101	1.43E − 118	3.94E − 120
82-68-8	Pentachloronitrobenzene	1.63E − 08	5.67E − 09	5.21E − 09	2.00E − 197	4.53E − 221	5.05E − 223
83-32-9	Acenaphthene	6.81E − 09	2.37E − 09	2.17E − 09	7.17E − 217	1.27E − 241	9.49E − 244
84-74-2	Dibutyl Phthalate	9.53E − 09	3.31E − 09	3.04E − 09	2.83E − 209	1.21E − 233	1.16E − 235
85-44-9	Phthalic Anhydride	6.53E − 09	2.27E − 09	2.07E − 09	7.88E − 218	1.16E − 242	6.83E − 245
86-73-7	Fluorene	3.19E − 07	1.11E − 07	1.02E − 07	4.40E − 138	6.38E − 158	1.44E − 159
87-86-5	Pentachlorophenol	2.28E − 08	7.91E − 09	7.26E − 09	3.42E − 190	1.81E − 213	2.05E − 215
91-20-3	Naphthalene	9.45E − 05	3.28E − 05	3.01E − 05	2.40E − 54	5.93E − 67	4.90E − 68
91-57-6	2-Methylnaphthalene	1.30E − 07	4.53E − 08	4.16E − 08	7.25E − 155	8.02E − 176	1.42E − 177
92-52-4	Biphenyl	3.81E − 07	1.32E − 07	1.21E − 07	7.06E − 135	1.42E − 154	3.00E − 156
92-87-5	Benzidine	4.55E − 08	1.58E − 08	1.44E − 08	9.87E − 176	4.20E − 198	3.95E − 200
94-75-7	2,4-Dichlorophenoxyacetic Acid	3.34E − 06	1.11E − 06	8.62E − 07	8.78E − 99	2.19E − 116	1.24E − 120
95-48-7	o-Cresol	3.18E − 09	1.11E − 09	1.01E − 09	1.36E − 234	3.06E − 260	1.32E − 262
95-95-4	2,4,5-Trichlorophenol	6.39E − 09	2.22E − 09	2.04E − 09	2.52E − 218	3.37E − 243	3.02E − 245
96-12-8	1,2-Dibromo-3-Chloropropane	4.41E − 07	1.53E − 07	1.41E − 07	2.88E − 132	9.51E − 152	2.62E − 153
96-45-7	Ethylene Thiourea	1.44E − 12	4.79E − 13	3.72E − 13	0.00E + 00	0.00E + 00	0.00E + 00
98-82-8	Cumene	3.10E − 07	1.08E − 07	9.88E − 08	1.33E − 138	1.87E − 158	3.42E − 160
98-86-2	Acetophenone	2.37E − 08	8.24E − 09	7.53E − 09	2.29E − 189	1.52E − 212	1.39E − 214

98-95-3	Nitrobenzene	3.19E − 06	1.11E − 06	1.01E − 06	1.73E − 99	2.19E − 116	5.80E − 118
	Total	8.23E − 03	2.82E − 03	2.42E − 03	5.43E − 16	5.23E − 23	4.16E − 24

a. Values of 0 are very small values approximated to 0.

Table S5. Rankings for emissions, Kow, toxicity values, and estimated risks for 95 organic chemicals analyzed in this study.

CASRN	Name	Ranking			
		Emission	ED_{50}, inhalation	ED_{50}, dietary ingestion	Estimated risk
100-41-4	Ethyl Benzene	7	92	76	20
100-42-5	Styrene	9	61	91	21
106-46-7	1,4-Dichlorobenzene	34	82	81	32
106-88-7	1,2-Epoxybutane	73	24	20	45
106-89-8	Epichlorohydrin	59	13	10	23
106-93-4	Ethylene Dibromide	64	73	69	52
106-99-0	1,3-Butadiene	10	34	30	53
107-02-8	Acrolein	12	1	2	1
107-05-1	Allyl Chloride	69	31	27	77
107-13-1	Acrylonitrile	35	15	12	24
107-21-1	Ethylene Glycol	8	86	86	81
108-05-4	Vinyl Acetate	27	49	45	37
108-10-1	Methyl Isobutyl Ketone	15	93	93	50
108-31-6	Maleic Anhydride	52	58	55	53
108-39-4	m-Cresol	71	68	64	84
108-88-3	Toluene	4	75	83	3
108-90-7	Chlorobenzene	30	72	68	28
108-95-2	Phenol	14	78	74	41
109-86-4	Ethylene Glycol Methyl E	46	54	50	82
110-54-3	Hexane	6	63	58	17
110-80-5	Cellosolve Solvent	79	71	67	87
114-26-1	Propoxur	94	23	19	87
117-81-7	Bis(2-Ethylhexyl)Phthala	43	47	42	30
118-74-1	Hexachlorobenzene	81	28	24	36
120-12-7	Anthracene	47	91	92	85
120-82-1	1,2,4-Trichlorobenzene	55	65	60	31
121-14-2	2,4-Dinitrotoluene	77	21	17	39
121-44-8	Triethylamine	48	55	51	66
121-69-7	N,N-Dimethylaniline	75	36	32	80
123-31-9	Hydroquinone	60	53	49	83
127-18-4	Tetrachloroethylene	17	51	47	2
129-00-0	Pyrene	29	67	63	55
133-06-2	Captan	40	60	57	48
133-90-4	Chloramben	95	42	37	87
1582-09-8	Trifluralin	20	41	36	15
1634-04-4	Methyl Tert-Butyl Ether	42	85	85	56
206-44-0	Fluoranthene	33	76	71	63
50-00-0	Formaldehyde	2	66	61	6
510-15-6	Chlorobenzilate	89	39	34	89
51-28-5	2,4-Dinitrophenol	78	40	35	72
532-27-4	2-Chloroacetophenone	82	2	1	26
542-75-6	1,3-Dichloropropene	44	3	33	4
56-23-5	Carbon Tetrachloride	49	14	11	9
56-38-2	Parathion	90	17	14	88
57-74-9	Chlordane	92	12	2	68
58-89-9	1,2,3,4,5,6-Hexachlorocy	87	11	9	60
593-60-2	Vinyl Bromide	86	32	28	87
62-53-3	Aniline	57	29	25	73
62-73-7	Dichlorvos	88	6	8	79
63-25-2	Carbaryl	32	42	37	38

67-56-1	Methanol	1	90	90	34
67-66-3	Chloroform	23	50	46	12
67-72-1	Hexachloroethane	62	19	15	14
68-12-2	N,N-Dimethylformamide	54	33	29	75
71-43-2	Benzene	5	74	70	5
71-55-6	Methyl Chloroform	13	94	94	33
72-43-5	Methoxychlor	91	58	55	87
74-83-9	Methyl Bromide	24	8	73	16
74-87-3	Methyl Chloride	21	64	59	49
75-00-3	Ethyl Chloride	39	95	95	86
75-01-4	Vinyl Chloride	31	38	7	35
75-05-8	Acetonitrile	26	79	75	58
75-07-0	Acetaldehyde	3	48	44	10
75-09-2	Methylene Chloride	19	52	48	19
75-15-0	Carbon Disulfide	18	18	62	13
75-25-2	Bromoform	67	57	53	44
75-35-4	Vinylidene Chloride	66	62	43	76
75-56-9	Propylene Oxide	37	20	16	22
76-44-8	Heptachlor	93	30	26	87
77-47-4	Hexachlorocyclopentadiene	76	4	31	7
78-59-1	Isophorone	51	87	87	74
78-87-5	Propylene Dichloride	63	10	6	11
79-00-5	1,1,2-Trichloroethane	56	27	23	18
79-06-1	Acrylamide	65	9	5	64
79-10-7	Acrylic Acid	50	5	79	59
79-46-9	2-Nitropropane	68	46	41	42
80-62-6	Methyl Methacrylate	22	35	84	29
82-68-8	Pentachloronitrobenzene	85	25	21	65
83-32-9	Acenaphthene	38	80	77	69
84-74-2	Dibutyl Phthalate	58	83	82	67
85-44-9	Phthalic Anhydride	53	89	89	70
86-73-7	Fluorene	28	76	71	46
87-86-5	Pentachlorophenol	80	45	40	62
91-20-3	Naphthalene	11	37	54	8
91-57-6	2-Methylnaphthalene	36	56	52	51
92-52-4	Biphenyl	41	68	64	43
92-87-5	Benzidine	74	16	13	57
94-75-7	2,4-Dichlorophenoxy Acet	16	44	39	25
95-48-7	o-Cresol	61	68	64	78
95-95-4	2,4,5-Trichlorophenol	72	81	78	71
96-12-8	1,2-Dibromo-3-Chloroprop	84	7	4	40
96-45-7	Ethylene Thiourea	83	26	22	87
98-82-8	Cumene	25	84	80	47
98-86-2	Acetophenone	45	88	88	61
98-95-3	Nitrobenzene	70	22	18	27
